# Successful treatment of iatrogenic internal carotid artery pseudoaneurysm following carotid endarterectomy with thrombin injection: a case report

**DOI:** 10.1186/s13256-024-04606-z

**Published:** 2024-06-18

**Authors:** Changchun Jiang, Jiahui Liu, Na Zhuo, Jianqi Wei, Yu Fan

**Affiliations:** 1 Department of Neurology, Baotou Central Hospital, No. 61 Huanchenglu, Donghe District, Baotou, 014040 Inner Mongolia China; 2 Neurointerventional Medical Center of Inner Mongolia Medical University, Baotou, Inner Mongolia China; 3 Neurological Diseases Clinical Medicine Research Center, Baotou, Inner Mongolia China; 4 Inner Mongolia Medical University, Hohhot, Inner Mongolia China

**Keywords:** Pseudoaneurysm, Carotid endarterectomy, Hybrid surgery, Thrombin, C-arm guidance

## Abstract

**Background:**

Iatrogenic pseudoaneurysms arising from the internal carotid artery subsequent to carotid endarterectomy are exceptionally infrequent. Herein, we present a case detailing an internal carotid artery pseudoaneurysm that manifested subsequent to a hybrid carotid endarterectomy and endovascular therapy intervention. Our approach to managing this condition involved a novel technique wherein thrombin was directly injected into the luminal cavity of the pseudoaneurysm under the guidance of a C-arm.

**Case presentation:**

A 66-year-old male patient of Chinese ethnicity exhibited a 4-month history of headache and a 20-day history of gait disturbance. Digital subtraction angiography revealed occlusion in the cervical region of the left carotid artery. Following a hybrid surgical procedure, the patient reported mild pain and bruising surrounding the incision site of the left internal carotid artery endarterectomy. Subsequent angiography identified the presence of a carotid artery pseudoaneurysm. Utilizing C-arm guidance, thrombin was then directly injected into the luminal cavity of the pseudoaneurysm, resulting in complete healing during follow-up.

**Conclusion:**

For the management of pseudoaneurysms arising post carotid endarterectomy, the direct injection of thrombin into the aneurysm cavity under the guidance of a C-arm is deemed both safe and efficacious.

## Introduction

Complications associated with carotid endarterectomy (CEA) encompass distal arterial embolism, cranial nerve injury, and cardiovascular accidents [1]. However, the occurrence of pseudoaneurysm within the carotid segment subsequent to CEA is remarkably uncommon, with a previously documented incidence ranging from 0.3% to 0.6% [2]. Established modalities for addressing pseudoaneurysms following CEA include surgical incision and endovascular interventions [3, 4]. Limited literature exists regarding the utilization of ultrasound-guided intraluminal thrombin injection for pseudoaneurysm treatment [5]. This report details a case involving an internal carotid artery pseudoaneurysm following a hybrid CEA and endovascular therapy procedure. The innovative approach employed in this case entailed the direct injection of thrombin into the luminal cavity of the pseudoaneurysm under the guidance of a C-arm. The ethics committee of Baotou Central Hospital approved the case report in compliance with the Helsinki Declaration. Written informed consent was obtained from the patient for publication of this case report and any accompanying images.

## Case report

A 66-year-old male of Chinese ethnicity presented with a 4-month history of headache and a 20-day history of gait disturbance. The initial neurological examination revealed right limb weakness, yielding a National Institutes of Health Stroke Scale (NIHSS) score of 1. Magnetic resonance imaging of the brain disclosed bilateral frontal lobe lacunar infarctions, with inadequate visualization of the left internal carotid and middle cerebral arteries. The patient had a 9-year history of diabetes mellitus, managed with insulin, maintaining blood glucose levels within the normal range. Notably, there was no history of hypertension, coronary heart disease, hypercholesterolemia, or smoking. Furthermore, there was no familial predisposition to the condition.

**Fig. 1:**
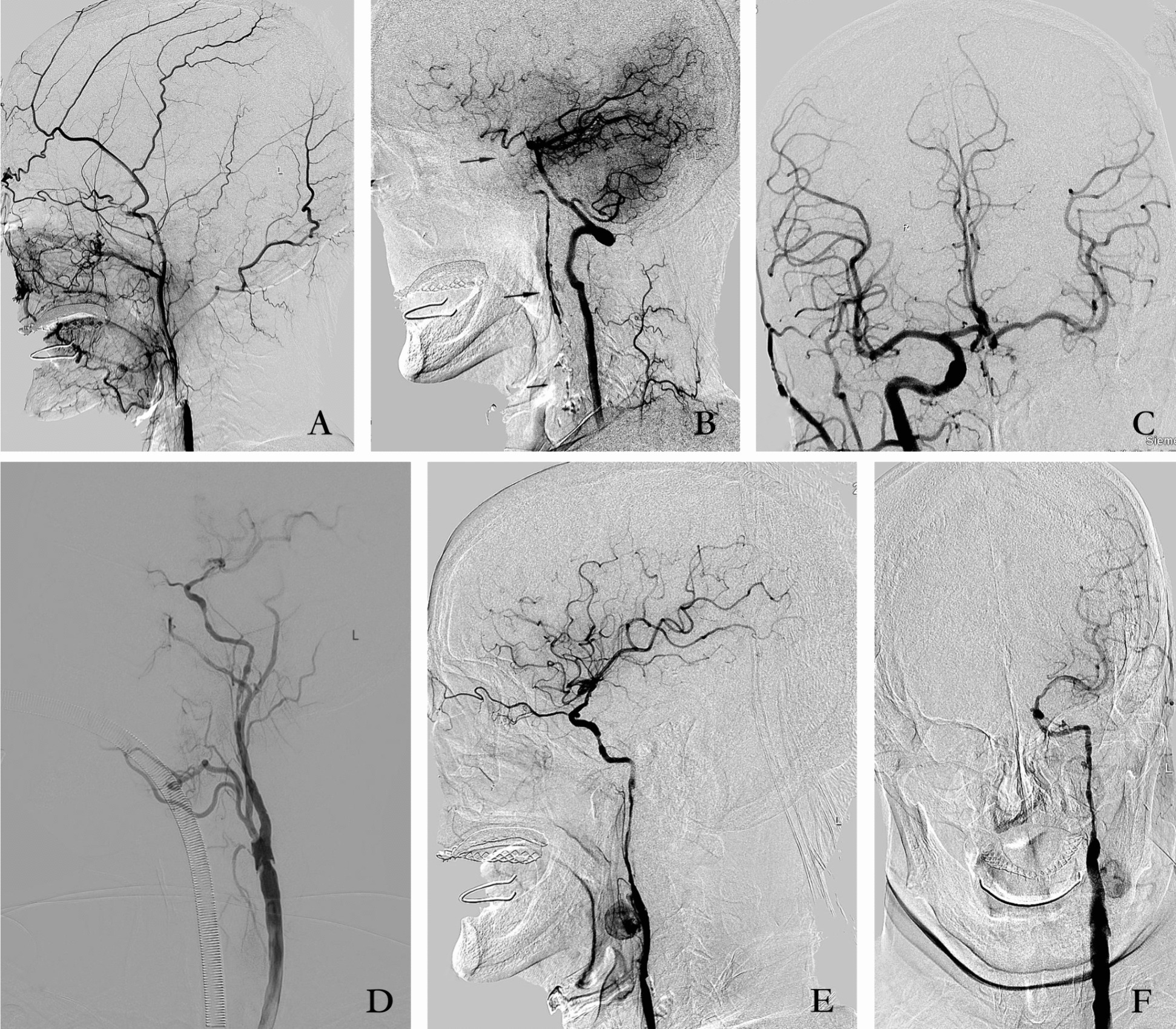
**A** Lateral angiogram of the left common carotid artery showing occlusion of the C1 segment of the left internal carotid artery. **B** Lateral angiogram of the left vertebral artery, showing the posterior communicating artery compensating for the distal C6 segment and the cervical muscular branch compensating for the C2 and C3 segments. **C** Frontal angiogram of the right common carotid artery showing partial compensation of the left middle cerebral artery flow by the anterior communicating artery. **D** Lateral angiography of the left internal carotid artery after a hybrid procedure showing revascularization of the left internal carotid artery. **E**, **F** An angiogram 10 days after the operation showing a 3.2 × 2.5 × 3.5 cm pseudoaneurysm of the left internal carotid artery

Admission digital subtraction angiography revealed an occlusion in the cervical segment of the left carotid artery, with the anterior communicating artery and posterior communicating arteries serving as secondary collaterals compensating for the intracranial blood supply to the left carotid artery (Fig. 1A–C). Given that conventional medical interventions failed to ameliorate symptoms associated with the symptomatic internal carotid artery occlusion, a standard carotid endarterectomy was executed.

Subsequently, the cavernous sinus segment of the internal carotid artery underwent angioplasty using a 2.0–15 mm balloon (Gateway) in a hybrid operating room equipped with an angiographic system. Repeat angiography after 20 minutes revealed continued perfusion of the internal carotid artery, and no stent placement was undertaken. Ultimately, complete recanalization of the left internal carotid artery was achieved without postanesthesia discomfort (Fig. 1D).

**Fig. 2:**
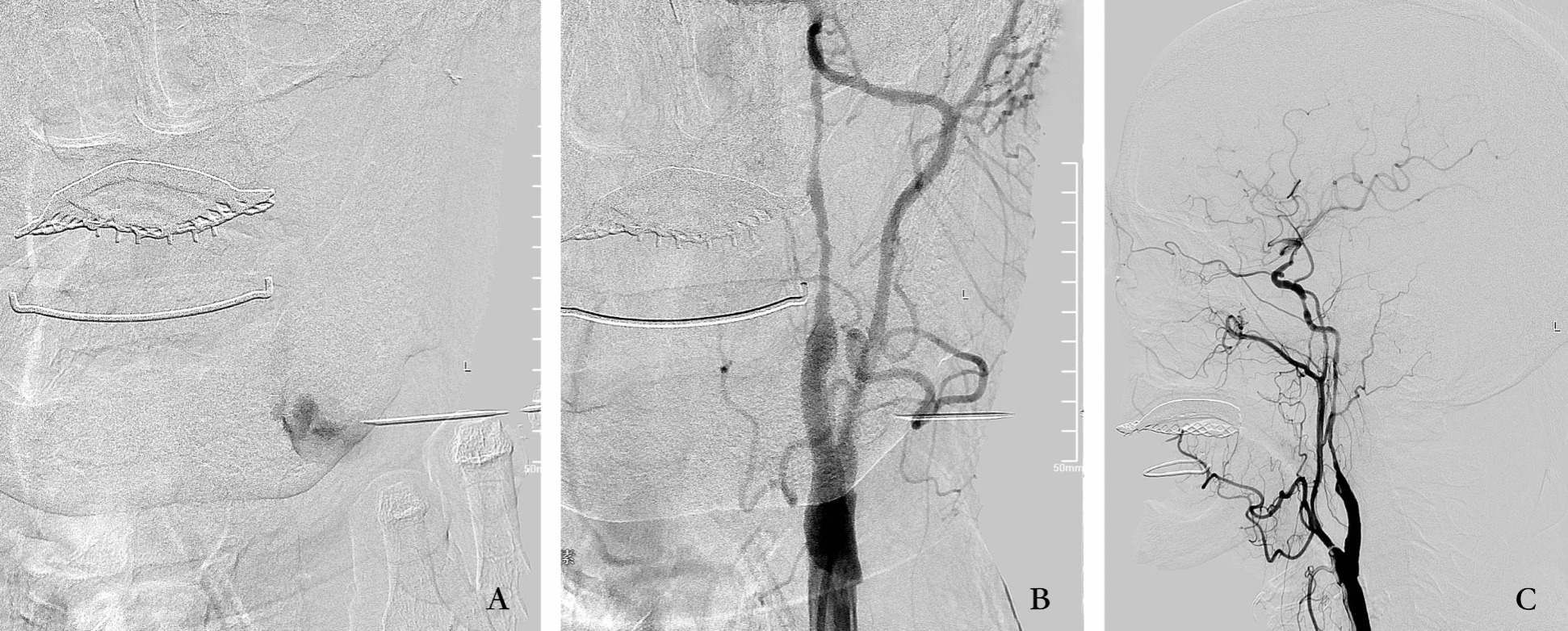
**A** A 22-gauge needle was punctured into the pseudoaneurysm sac, and a small amount of contrast was injected to show the outline of the pseudoaneurysm. **B**, **C** Thrombin (150 U) was injected into the sac of the pseudoaneurysm, the angiogram showed complete occlusion of the pseudoaneurysm with normal internal carotid flow and no distal embolic events

Postoperatively, the patient gradually developed mild pain and swelling at the left internal carotid endarterectomy incision. The symptoms were not relieved by medication. An angiogram performed 10 days after the operation revealed a pseudoaneurysm measuring 3.2 × 2.5 × 3.5 cm, with a neck extending from the cervical segment of the left internal carotid artery (Fig. 1E, F). Our initial plan to address this with a covered stent was rendered impracticable owing to the unavailability of a covered stent of appropriate diameter for the location. Consequently, the decision was made to manage the pseudoaneurysm through the direct puncture injection of thrombin, drawing upon our experience with femoral artery pseudoaneurysms. Informed consent was obtained for both diagnostic angiography and percutaneous treatment. A 22-gauge needle was employed to puncture the pseudoaneurysm under C-arm guidance, with frontal and lateral positioning ensuring precision, and pulsatile blood flow was subsequently observed upon needle insertion. Confirmation of needle entry into the pseudoaneurysm sac was achieved by injecting a small amount of contrast through the needle (Fig. 2A). Following this confirmation, thrombin (150 units) was injected into the pseudoaneurysm sac. Subsequent angiography exhibited complete occlusion of the pseudoaneurysm, restoring normal internal carotid flow without distal embolic events (Fig. 2B, C). Postprocedure, the patient remained asymptomatic, and local bruising and pain resolved. A repeated carotid ultrasound performed 2 days later confirmed the occlusion of the pseudoaneurysm. A follow-up angiogram conducted 2 months later demonstrated normal blood flow in the left internal carotid artery, with no visualization of the pseudoaneurysm, indicating complete occlusion.

## Discussion

The hybrid surgical revascularization of chronic occlusion in the internal carotid artery has recently gained acceptance as a more conventional procedure, demonstrating a notable success rate in achieving reperfusion, particularly in cases involving tandem occlusion of the carotid artery [6]. Pseudoaneurysm formation subsequent to CEA represents a relatively infrequent complication. In the case series presented by Abdelhamid, the incidence of pseudoaneurysm post-CEA was reported as 0.4% (5/12,000) [7]. Among the subset of patients developing pseudoaneurysms, all underwent angioplasty with a patch during the initial CEA procedure [7]. Reports indicate that pseudoaneurysm rates are two to four times more prevalent when patches are used in CEA procedures compared with direct closure of the vessel [8]. This higher occurrence may be attributed to an elevated incidence of infections associated with patch usage. Additional factors correlated with pseudoaneurysm development following CEA include bacteremia, postoperative hypertension, insufficient haemostasis, and arterial wall or patch dilation [7, 9].

In this specific case, direct vascular closure was employed, and no postoperative infection evidence was discerned. The pseudoaneurysm could have arisen owing to the patient’s fragile arterial wall, where excessive tension during closure resulted in a postoperative rupture, leading to pseudoaneurysm formation. Conversely, during endovascular treatment, the guidewire traverses meticulously over the upper edge of the carotid artery endarterectomy site, and the balloon does not necessitate angioplasty at the corresponding site of the pseudoaneurysm. Hence, the endovascular procedure is not implicated in pseudoaneurysm formation. The pseudoaneurysm in our patient was identified shortly after the procedure, presenting only with local swelling and bruising. Unlike previously reported cases in literature, there was no manifestation of ischemic events, intracranial arterial embolism, direct compression of local neck structures, or rupture leading to hematoma [10].

Traditional approaches for managing pseudoaneurysms following CEA involve open surgical procedures, encompassing aneurysm resection with end-to-end anastomosis, aneurysm resection with patchplasty, carotid artery bypass grafting, and carotid artery ligation. While these surgical interventions afford direct excision of the aneurysm, vascular restoration, elimination of infected local tissue, and reduced recurrence, open surgery is accompanied by a notable incidence of surgical complications. A systematic review conducted by Heskett *et al*. revealed that open surgery exhibited an almost 30% rate of procedure-related complications [11]. In recent years, with advancements in interventional techniques and materials, endovascular treatment has emerged as an effective modality for addressing pseudoaneurysms post-CEA, particularly in cases of noninfectious pseudoaneurysms. Endovascular treatment demonstrates a lower complication rate compared with open surgery. In the early stages, bare stents with or without coil embolization are commonly used, while covered stents for angiogenesis are more useful in the later stages of pseudoaneurysms after CEA [7, 12].

Thrombin is frequently administered to manage iatrogenic pseudoaneurysms resulting from femoral artery puncture during interventional therapy. Kang *et al*. documented 83 cases of thrombin injections for pseudoaneurysms, with 73 involving the femoral artery. Initial therapies were successful following thrombin injections, with seven cases experiencing recurrence necessitating additional treatment. This early report, encompassing a substantial patient cohort, establishes the safety and reliability of thrombin in treating peripheral pseudoaneurysms [13]. Holder *et al*. initially reported a case of a common carotid artery pseudoaneurysm resulting from internal jugular vein cannulation, which was successfully treated with ultrasound-guided injection of 250 units of thrombin into the pseudoaneurysm lumen under the protection of an occlusion balloon. Subsequently, Lee *et al*. reported a similar case of a common carotid artery pseudoaneurysm post-jugular venipuncture, treated with a 200-unit thrombin injection under the protection of a distal embolic protection device. A 2 mm thrombus was identified within the distal embolic protection device, and the authors attributed its formation to thrombin injection [5]. However, they noted the placement of the protective device in the proximal bifurcation of the common carotid artery, while the patient exhibited plaque at the beginning of the internal carotid artery, suggesting other potential causes of thrombosis, such as the device itself [14].

In our case, the treatment approach involved the application of direct thrombin injection for the pseudoaneurysm, and a protective device was not employed. This decision was influenced by our prior experience with thrombin treatment for femoral artery pseudoaneurysms and instances of ruptured bleeding during the embolization of intracranial aneurysms. For femoral artery pseudoaneurysms, we utilize a thrombin concentration of 100 μ/ml, administering 0.5–1 ml of thrombin into the pseudoaneurysm cavity under ultrasound guidance. Immediate thrombus formation is observed within the pseudoaneurysm cavity without distal vascular embolism. This phenomenon is likely owing to the pseudoaneurysm’s relatively narrow neck, facilitating rapid thrombus formation upon contact with stagnant blood, as thrombin does not directly bind to blood in the vessel to induce clotting.

In our previous reports, we detailed two cases of aneurysmal hemorrhage during aneurysm coiling, necessitating direct thrombin injection into the aneurysm cavity after failed therapeutic interventions. In both instances, prompt hemostasis was achieved with minimal adverse outcomes, and no distal embolic events occurred following thrombin injection [15]. Adhering to established clinical practice, a protective device was not utilized to prevent distal arterial embolism in the case of the carotid pseudoaneurysm. However, it is essential to underscore that, when administered at the appropriate dosage and concentration, thrombin remains an effective safeguard against potential distal embolism.

Previous studies have used ultrasound guidance to identify the tip of the needle in the pseudoaneurysm cavity before injecting thrombin, whether in the femoral artery or other sites of the pseudoaneurysm. However, in many instances, it is difficult and time-consuming to locate the needle by ultrasound. In this case, after angiography using the C-arm had confirmed the presence of a carotid artery pseudoaneurysm, the needle was located directly in the cavity of the pseudoaneurysm under frontal and lateral fluoroscopic positioning of the C-arm. In contrast, the needle was injected with contrast into the cavity of the pseudoaneurysm, improving the safety of this procedure by visually confirming that the needle was in the cavity of the pseudoaneurysm. Another advantage of C-arm guidance is the ability to perform a whole brain angiogram after thrombin injection to monitor intracranial arterial blood flow and treat distal embolic events at the first opportunity if they occur. Therefore, it may be more appropriate to use C-arm positioning when performing thrombin injections for carotid pseudoaneurysms.

## Conclusion

For the management of pseudoaneurysms arising post carotid endarterectomy, the direct injection of thrombin into the aneurysm cavity under the guidance of a C-arm is deemed both safe and efficacious. Prompt postoperative symptom monitoring, and ultrasound examination of the neck vessels can detect pseudoaneurysms at an early stage and treat them as soon as possible.

## Data Availability

Not applicable.
